# Antimicrobial therapy and assessing therapeutic response in culture-negative pyogenic vertebral osteomyelitis: a retrospective comparative study with culture-positive pyogenic vertebral osteomyelitis

**DOI:** 10.1186/s12879-020-05669-1

**Published:** 2020-12-09

**Authors:** Dongwoo Yu, Sang Woo Kim, Ikchan Jeon

**Affiliations:** Department of Neurosurgery, Yeungnam University Hospital, Yeungnam University College of Medicine, 170, Hyeonchung street, Nam-Gu, Daegu, 42415 South Korea

**Keywords:** Vertebral osteomyelitis, Pyogenic, Negative culture, Antibiotics, Lumbar

## Abstract

**Background:**

There are still controversies regarding the treatment and outcomes in culture-negative pyogenic vertebral osteomyelitis (PVO). The purpose of this study is to investigate the antimicrobial therapy, assessment of therapeutic response, and outcome of culture-negative PVO compared to culture-positive PVO.

**Methods:**

A retrospective study was performed with non-surgical lumbar PVO patients. The patients were divided into two groups based on the causative bacterial identification (CN group with culture-negative PVO and CP group with culture-positive PVO). The clinical features, use of antibiotics, laboratory data, and outcomes were compared between the two groups.

**Results:**

Seventy-three patients with 41 (56.2%) of the CN group and 32 (43.8%) of the CP group were enrolled. The CN group showed a shorter duration of parenteral antibiotics (45.88 ± 16.14 vs. 57.31 ± 24.39, *p* = 0.019) but a tendency of prolonged duration of total (parenteral + oral) antibiotics (101.17 ± 52.84 vs. 84.19 ± 50.29 days, *p* = 0.168). When parenteral antibiotics were discontinued or switched to oral antibiotics, the mean erythrocyte segmentation rate (ESR, normal range: < 25 mm/h), C-reactive protein (CRP, normal range: < 0.5 mg/dL) level, and visual analog scale (VAS) score of back pain were 42.86 ± 24.05 mm/h, 0.91 ± 1.18 mg/dL, and 4.05 ± 1.07, respectively, with no significant differences between the two groups. The recurrence rates of CN and CP groups were 7.3% (3/41) and 6.3% (2/32), respectively (*p* = 1.000). The presence of epidural abscess was the most significant factor for the identification of causative bacteria (*p* = 0.002), and there was no significant relationship between the use of empirical antibiotics before tissue culture and the causative bacterial identification (*p* = 0.194).

**Conclusions:**

The CN group required a shorter duration of parenteral antibiotics than the CP group. Discontinuation of parenteral antibiotics or changing the administration route can be considered based on the values of ESR, CRP, and VAS score of back pain. The presence of epidural abscess was the most significant factor for the identification of causative bacteria.

## Background

Vertebral osteomyelitis is an infectious disease that can develop by pyogenic, tuberculous, or brucellar causes [1]. The annual incidence of hospitalization with pyogenic vertebral osteomyelitis (PVO) in the United States between 1998 and 2013 rose from 2.9 to 5.4 per 100,000 individuals [2]. There is still no clear guidance regarding the duration and route of antibiotic administration. Generally, an extended course of parenteral antibiotics followed by a maintenance course of oral antibiotics is required in the treatment of PVO [3–5]. Infectious Diseases Society of America (IDSA) recommends 6 weeks of parenteral or highly bioavailable oral antimicrobial therapy for bacterial native vertebral osteomyelitis [6].

Identification of the causative bacteria and the use of appropriate antibiotics are necessary to achieve successful treatment and favorable outcomes in PVO. Despite a lot of effort, it is not always possible to identify the causative microorganism in clinical practice. About 50% of patients with PVO are treated with empirical antibiotics because PVO often shows negative in the microbiological culture test [7, 8]. The guidelines for the choice and preferred route of antibiotic administration for culture-negative PVO have not been well established owing to insufficient relevant research result, which is originated in the variety of causative microorganisms and rate of antibiotic resistance depending on the regions. Herein, we conducted a retrospective study to compare culture-negative PVO and culture-positive PVO in terms of clinical characteristics, use of antibiotics, assessment of therapeutic response, and outcomes.

## Methods

### Patients and data collection

This retrospective study was performed at a single tertiary-care university hospital from October 2014 to September 2018 and included 118 patients diagnosed as pyogenic lumbar PVO. Patients were excluded if they had tuberculous spondylitis, tumors, postoperative development, accompanying bone infection at another site, trauma, pregnancy, concomitant severe medical problems, less than six months of follow-up period, or age < 19 years. All clinical and radiological data were obtained and reviewed retrospectively from electronic charts under the approval of the institutional review board (Yeungnam University Hospital, 2020-06-091).

### Diagnosis of PVO

PVO was diagnosed based on clinical symptoms, laboratory data, and radiological findings. The clinical symptoms included fever and back pain with or without radiating pain. Elevated erythrocyte segmentation rate (ESR, normal range: < 25 mm/h) or C-reactive protein (CRP, normal range: < 0.5 mg/dL) level or both along with specific magnetic resonance (MR) findings of PVO as a contiguous single lesion was an important clue for the diagnosis of PVO. In the extent of PVO lesion, a PVO lesion comprises the upper and lower vertebrae centering on the infected disc with or without epidural, psoas, and paraspinal abscesses; it was defined as one level (e.g., if there were two infected discs, then three vertebrae were included centering on the two infected discs, this PVO lesion was considered two levels) [9] .

### Causative bacterial identification

Patients diagnosed as PVO were divided into two groups according to the results of causative bacterial identification. The microbiological diagnosis was confirmed through more than two sets of blood culture or tissue culture for the PVO lesion by computed tomography (CT)-guided needle biopsy or open surgical biopsy [10]. Patients with clinical symptoms and radiological findings indicative of PVO but without a confirmed causative bacterial identification were defined as the culture-negative group (CN group), and other patients in whom the causative microorganism was identified were defined as the culture-positive group (CP group).

### Cure and recurrence

The patients underwent clinical assessment for therapeutic response after at least three weeks of parenteral antibiotic therapy, which was performed based on clinical symptoms (fever and back pain) and hematological inflammatory indices (CRP and ESR). The condition of being ‘Cured’ was defined as the absence of fever and resolution in back pain and improvement of CRP level, which continued during a follow-up period of at least six months without any other evidence of residual or recurrence of PVO. When a patient was determined as ‘Cured’ on clinical assessment for therapeutic response, antibiotic therapy was discontinued. The condition of being ‘Non-cured’ was defined as follows: persistence or re-aggravation of clinical symptoms and hematological indicies with the presence of fever and/or re-identification of same causative bacteria from blood/PVO lesion and the development of new or re-aggravation of the PVO lesion on MR imaging after discontinuation of antibiotic therapy within the follow-up period of at least six months [11, 12].

### Functional and laboratory evaluations

All patients underwent the evaluation of clinical status and laboratory tests for the initial three months after the diagnosis of PVO and on starting antibiotic therapy. Visual analog scale (VAS) scores for back pain and laboratory data, including ESR and CRP at initial (at the time of diagnosis), 1 week, 1 month, and 3 months were compared between the two groups. VAS was used to estimate back pain, with 0 representing no pain to 10 representing maximum pain. The values of ESR and CRP when parenteral antibiotics were discontinued or switched to oral antibiotics were measured and compared between the two groups.

### Statistical analysis

The Student’s t-test and the Mann-Whitney U test were used to compare parametric and non-parametric continuous variables, respectively. One-way analysis of variance (ANOVA) for parametric continuous variables and the Kruskal-Wallis test for non-parametric continuous variables were used to compare three population means. For categorical variables, the chi-square test was performed. Logistic regression analysis was used to investigate the clinical variables related to the identification of causative microorganisms. All clinical variables identified as statistically significant in univariate analyses were included in a multivariate analysis. Statistical analysis was carried out using SPSS version 25.0 software (SPSS Inc., Chicago, Illinois), and *p* values of < 0.05 were considered statistically significant.

## Results

### Demographic and clinical data

Among the 118 patients, 45 were excluded due to surgery-related development (*n =* 25), tuberculosis (*n =* 12), ankylosing spondylitis (*n =* 2), and follow-up loss (*n =* 6). The final analyses were performed on 73 patients (44 men and 29 women) with a mean age of 64.73 ± 11.61 (42-84) years. There were no statistically significant differences in age, gender, underlying diseases, and history of spinal procedures between the two groups. However, there were statistically significant differences in the extent of affected PVO lesion [1.49 ± 0.93 (1-5) vs. 2.09 ± 1.28 (1-5) levels, *p* = 0.022] and the presence of epidural abscess (43.9% [18/41] vs. 84.4% [27/32], *p* = 0.001), except the presence of psoas muscle and paraspinal abscess. The recurrence rates were 7.3% (3/41) and 6.3% (2/32), respectively (*p* = 1.000). Detailed data are mentioned in Table 1.

**Table 1:** Demographic and clinical data

	CN group (*n =* 41)	CP group (*n =* 32)	*p* value ^a^	Total (*n =* 73)
Age [year]	65.41 ± 11.60	61.83 ± 13.76	0.570	64.73 ± 11.61 (42-84)
Male sex	27 (65.9%)	17 (53.1%)	0.337	44 (60.3%)
BMI	22.97 ± 3.25	24.27 ± 3.75	0.120	23.54 ± 3.52
Underlying disease				
Diabetes mellitus	14 (34.1%)	7 (21.9%)	0.304	21 (28.8%)
Rheumatic disease	3 (7.3%)	1 (3.1%)	0.626	4 (5.5%)
Liver cirrhosis	2 (4.9%)	0 (0%)	0.501	2 (2.7%)
Chronic kidney disease	1 (2.4%)	0 (0%)	1.000	1 (1.4%)
Alcohol	13 (31.7%)	4 (12.5%)	0.092	17 (23.3%)
Smoking	15 (36.6%)	6 (18.8%)	0.121	21 (28.8%)
History of spinal procedures	27 (65.9%)	18 (56.3%)	0.471	45 (61.6%)
Epidural injection	25 (55.6%)	18 (50.0%)	0.659	43 (53.1%)
Acupuncture [oriental medicine]	10 (24.4%)	4 (12.5%)	0.242	14 (19.2%)
Fever [°C, > 37.3]	11 (26.8%)	8 (25.0%)	1.000	19 (26.0%)
Neurological symptom	17 (41.5%)	20 (62.5%)	0.100	37 (50.7%)
Features of PVO				
Extent of affected lesion [level]	1.49 ± 0.93 (1-5)	2.09 ± 1.28 (1-5)	0.022	1.75 ± 1.13 (1-5)
Epidural abscess	18 (43.9%)	27 (84.4%)	0.001	45 (61.6%)
Psoas abscess	17 (41.5%)	13 (40.6%)	1.000	30 (41.1%)
Paraspinal abscess	27 (65.6%)	23 (71.9%)	0.612	50 (68.5%)
Duration of hospital stay [days]	50.32 ± 19.96 (21-120)	62.88 ± 26.04 (26-143)	0.022	55.82 ± 23.51 (21-143)
Follow up period [months]	18.00 ± 11.16 (6-42)	16.38 ± 12.48 (6-63)	0.560	17.29 ± 11.70 (6-63)
Recurrence	3 (7.3%)	2 (6.3%)	1.000	5 (6.8%)

Data are presented as the mean ± standard deviation or frequency, *CN* culture negative, *CP* culture positive, *BMI* body mass index, *PVO* pyogenic vertebral osteomyelitis, ^a^ *p* value between group CN and CP, *p* values of < 0.05 were considered statistically significant.

### Microorganisms and antibiotics

The rate of causative bacterial identification was only 43.8% (32/73) with 8 (25%) from blood culture, 15 (46.9%) from tissue culture of CT-guided needle or open surgical biopsy of the PVO lesion, and 9 (28.1%) from both blood and tissue cultures. According to the culture methods, 17 of 73 patients (23.3%) with blood cultures, 7 of 37 patients (18.9%) with CT-guided needle biopsy, and 17 of 36 patients (47.2%) with open surgical biopsy were identified. Microbiologic findings of the 32 patients in the CP group are shown in Table 2. The most common causative microorganism was *Staphylococcus aureus* (40.6%, 13/32). Five cases of recurrence were noted: one with methicillin-resistant *Staphylococcus epidermidis,* one with *Enterobacter*, and three with culture-negative cases. Detailed data are mentioned in Table 2.

**Table 2:** Microbiologic findings

Factors	Values
Causative bacterial identification	32/73 (43.8%)
Blood culture	8 (25.0%)
Tissue culture of CT-guided needle biopsy on PVO lesion	5 (15.6%)
Tissue culture of open biopsy on PVO lesion	10 (31.3%)
Both (blood and tissue cultures)	9 (28.1%)
Positive culture rates depending on the methods	
Blood culture	17/73 (23.3%)
Tissue culture of CT-guided needle biopsy on PVO lesion	7/37 (18.9%)
Tissue culture of open biopsy on PVO lesion	17/36 (47.2%)
Microbiological findings (*n =* 32)	
Gram-positive bacteria	26 (81.3%)
*Staphylococcus aureus*	13 (40.6%)
MSSA	10 (31.2%)
MRSA	3 (9.4%)
*Coagulase-negative staphylococci*	4 (12.5%)
*Streptococcus* species	6 (18.8%)
*Streptococcus agalactiae*	2 (6.3%)
Others^a^	4 (12.5%)
*Enterococcus* species	3 (9.4%)
Gram-negative bacteria	6 (18.8%)
*Escherichia coli*	2 (6.3%)
*Klebsiella pneumoniae*	1 (3.1%)
*Acinetobactor baumannii*	2 (6.3%)
*Enterobacter* species	1 (3.1%)

Data are presented as frequency, PVO pyogenic vertebral osteomyelitis, MSSA methicillin-sensitive *Staphylococcus aureus*, MRSA methicillin-resistant *Staphylococcus aureus*, ^a^ *Streptococcus viridians, Streptococcus pneumoniae,* and *Streptococcus bovis*

The antibiotic regimens for the treatment of PVO are summarized in Table 3. β-Lactam and glycopeptide were the mainly used as the effective antibiotics in both groups. The use of glycopeptide did not significantly differ between the two groups. However, quinolones were used statistically significantly more in the CN group (22.0% [9/41] vs. 3.1% [1/41], *p* = 0.036). Antibiotics were administered before tissue culture of the PVO lesion in 71.2% cases (52/73), and there was no significant difference between the two groups (78.0% [32/41] vs. 62.5% [20/32], *p* = 0.194). There was no statistically significant difference in the duration of total antibiotics between the two groups (101.17 ± 52.84 of CN group vs. 84.19 ± 50.29 days of CP group, *p* = 0.168). However, there were statistically significant differences in the duration of parenteral antibiotics (45.88 ± 16.14 vs. 57.31 ± 24.39, *p* = 0.019), oral antibiotics (55.29 ± 47.40 vs. 26.84 ± 41.10, *p* = 0.009), and the incidence of using oral antibiotics (75.6% [31/41] vs. 43.8% [14/32], *p* = 0.001) between the two groups, respectively. Detailed data are provided in Table 4.

**Table 3:** Regimens of antibiotics

	CN group (*n =* 41)	CP group (*n =* 32)	*p* value ^#^	Total (*n =* 73)
Parenteral antibiotics	41 (100%)	32 (100%)	–	73 (100%)
β-Lactam	15 (36.6%)	11 (34.4%)	1.000	26 (35.6%)
1st generation cephalosporin	10 (24.4%)	3 (9.4%)	0.128	13 (17.8%)
3rd generation cephalosporin	4 (9.8%)	2 (6.3%)	0.689	6 (8.2%)
Nafcillin	1 (2.4%)	6 (18.8%)	0.039	7 (9.6%)
β-Lactam ± others ^a^	6 (14.6%)	5 (15.6%)	1.000	11 (15.1%)
Glycopeptide ± others ^b^	11 (26.8%)	12 (37.5%)	0.447	23 (31.5%)
Quinolone	9 (22.0%)	1 (3.1%)	0.036	10 (13.7%)
Others ^c^	0 (0%)	3 (9.4%)	0.080	3 (4.1%)
Oral antibiotics	33 (77.8%)	12 (38.9%)	0.001	49 (60.5%)
β-Lactam	7 (17.1%)	1 (3.1%)	0.072	8 (11.0%)
Quinolone	26 (63.4%)	11 (34.4%)	0.019	37 (50.7%)

*CN* culture negative, *CP* culture positive, Data are presented as frequency, ^a^ β-lactamase inhibitor, aminoglycoside, ^b^ Gentamicin and tazime, ^c^ Carbapenem, prepenem, and linezoid, ^#^ *p* value between the CN and CP groups, *p* values of < 0.05 were considered statistically significant

**Table 4:** Comparison of antibiotic treatment between the CN and CP groups

	CN group (*n =* 41)	CP group (*n =* 32)	*p* value ^#^	Total (*n =* 73)
Use of antibiotics before tissue-culture	32/41(78.0%)	20/32 (62.5%)	0.194	52/73 (71.2%)
Duration of antibiotic treatment (days)				
Total [parenteral + oral]	101.17 ± 52.84 (31-209)	84.19 ± 50.29 (25-198)	0.168	93.73 ± 52.08 (25-209)
Parenteral	45.88 ± 16.14 (17-95)	57.31 ± 24.39 (25-144)	0.019	50.89 ± 20.82 (17-144)
Oral	55.29 ± 47.40 (0-166)	26.84 ± 41.10 (0-126)	0.009	42.82 ± 46.66 (0-166)
Incidence of using oral antibiotics	31/41(75.6%)	14/32, (43.8%)	0.001	45/73 (61.6%)

Data are presented as the mean ± standard deviation or frequency, *CN* culture negative, *CP* culture positive, ^#^ *p* value between group CN and CP, *p* values of < 0.05 were considered statistically significant

The clinical variables related to the causative bacterial identification are presented in Table 5. The clinical variables with statistical significance identified in a univariate logistic regression analysis were included in a multivariate logistic regression analysis. Epidural abscess and initial VAS score of back pain were statistically significant predictors.

**Table 5:** Logistic regression analysis in the correlation between the causative bacterial identification (culture positive) and variable clinical variables

Clinical variable	Univariate			Multivariate		
	OR	95% CI	*p* value	OR	95% CI	*p* value
Open surgical biopsy	3.31	1.26-8.71	0.015			
Extent of affected lesion	1.65	1.06-2.58	0.027			
Epidural abscess	6.90	2.22-21.49	0.001	6.57	2.00-21.57	0.002*
Initial CRP	1.08	1.02-1.14	0.009			
Initial back VAS	2.00	1.21-3.33	0.007	1.95	1.13-3.38	0.017*

*OR* odds ratio, *CI* confidential interval, *CRP* C-reactive protein (normal range < 0.5 mg/dL), VAS visual analog scale, *p* values of < 0.05 were considered statistically significant

### Changes of ESR/CRP and VAS score for back pain during the early 3-month after starting antibiotics

There were statistically significant improvements in ESR, CRP, and VAS score of back pain in both groups during early 3 months, respectively (*p* < 0.01). In ESR, there were no statistically significant differences at initial, 1 week, 1 month, and 3 months findings between the two groups. However, there were statistically significant differences in CRP (9.21 ± 7.43 vs. 15.17 ± 10.32 mg/dL, *p* = 0.005) and VAS score of back pain (7.41 ± 0.92 vs. 8.13 ± 1.13, *p* = 0.004) at initial assessment between the two groups, respectively. However, there were no statistically significant differences in CRP and ESR at 1-week, 1-month, and 3-month between the two groups, respectively. When parenteral antibiotics discontinued or changed to oral antibiotics, there were no statistically significant differences in ESR, CRP, and VAS score of back pain between the two groups. The values of ESR, CRP, and VAS score of back pain in total patients were 44.85 ± 24.99 mm/h, 0.89 ± 1.02 mg/dL, and 3.98 ± 1.08, respectively. Detailed data are provided in Table 6.

**Table 6:** Changes of ESR/CRP and VAS score of back pain during 3 months since antimicrobial therapy started

	CN group (*n =* 41)	CP group (*n =* 32)	*p* value ^#^	Total (*n =* 73)
ESR				
Initial (at diagnosis)	66.88 ± 31.89	72.44 ± 31.89	0.462	69.32 ± 31.79
1 week	70.98 ± 33.81	74.31 ± 26.84	0.649	72.44 ± 30.79
1 month	55.29 ± 29.94	61.31 ± 31.07	0.405	57.93 ± 30.37
3 months	30.51 ± 19.65^+^	42.81 ± 31.44^+^	0.058	35.90 ± 26.03
CRP				
Initial (at diagnosis)*	9.21 ± 7.43	15.17 ± 10.32	0.005	11.83 ± 9.24
1 week	5.67 ± 5.75	6.97 ± 5.74	0.339	6.24 ± 5.74
1 month	1.89 ± 2.05	2.26 ± 2.75	0.516	2.06 ± 2.37
3 months	0.58 ± 0.99^+^	0.79 ± 1.29^+^	0.427	0.67 ± 1.13
VAS score of back pain				
Initial (at diagnosis)*	7.41 ± 0.92	8.13 ± 1.13	0.004	7.73 ± 1.07
1 week	5.73 ± 1.18	5.63 ± 1.39	0.724	5.68 ± 1.27
1 month	4.32 ± 1.13	4.47 ± 1.27	0.591	4.38 ± 1.19
3 months	3.37 ± 0.92^+^	3.44 ± 1.27^+^	0.780	3.39 ± 1.08
When discontinuation of parenteral antibiotics				
ESR	44.85 ± 24.99	40.31 ± 22.91	0.427	42.86 ± 24.05
CRP	0.89 ± 1.02	0.93 ± 1.37	0.907	0.91 ± 1.18
VAS score of back pain	3.98 ± 1.08	4.16 ± 1.05	0.476	4.05 ± 1.07

*CN* culture negative, *CP* culture positive, *ESR* erythrocyte segmentation rate (normal range < 25 mm/h), *CRP* C-reactive protein (normal range < 0.5 mg/dL), *VAS* visual analogue scale, ^#^ *p* value between group CN and CP, * There is statistical significant difference between the two groups (*p* < 0.01) ^+^, There is statistical significant difference compared to initial value (*p* < 0.01), *p* values of < 0.05 were considered statistically significant

## Discussion

The rate of causative bacterial identification varies as reported in the previous literatures [7, 11, 13], and this study showed lower rate with less than 50% compared to other reports. The result is expected to be the most relevant to the use of empirical antibiotics before tissue culture for the PVO lesion (52/73, 71.2%) due to poor general conditions, including high fever or sepsis, although blood cultures were performed in all patients before administering empirical antibiotics. However, there was no statistically significant relationship between the use of empirical antibiotics before tissue culture and the causative bacterial identification (32/41 in CN group vs. 20/32 in CP group, *p* = 0.194). There are still debates on the influence of empirical antibiotics before tissue culture on the microbiological diagnostic yield [14–17]. In addition, the rate of causative bacterial identification in PVO varies with the method of culture. Generally, the rate of positive-culture is higher with an open surgical biopsy than with needle biopsy, which is related to the obtaining sufficient specimen quantity from the accurate area of the infectious lesion. In multivariate logistic regression analysis, the presence of epidural abscess was the most powerful statistically significant predictor of causative bacterial identification. PVOs with epidural abscess were more likely to present neurological deficits such as leg pain or weakness. These may require a surgical treatment for removing epidural abscess to relieve neurological symptoms, which also allows obtaining a sufficient quantity of proper specimen, thus leading to increased microbiological diagnostic yield despite the use of empirical antibiotics in advance.

In laboratory data, the initial CRP level was significantly lower in the CN group than in the CP group. Previous studies reported fewer infectious signs and lower values of inflammatory markers in patients with culture-negative PVO, which may have been due to small inocula of pathogens [11, 13, 18]. Kim et al. [18] showed that culture-positive PVO is more likely to be associated with body temperature ≥ 37.8 °C, higher initial ESR and CRP, and the presence of paraspinal abscess. In this study, similar results were presented with a larger extent of the affected lesion, presence of epidural abscess, worse back pain, and higher initial CRP level in the CP group. Among the regimens of antibiotics, more frequent use of quinolone as an effective antibiotic in the CN group also supports these results. We performed serial follow-up of ESR, CRP, and VAS score of back pain. By the 3rd month, the CRP level had almost normalized and VAS scores had decreased by more than 50% of the initial scores. However, despite a tendency of continuous decline, the value of ESR remained consistently higher than normal. There were no statistically significant differences in ESR, CRP, and VAS score of back pain at 3 months between the two groups. We assume that the mechanical stress on the injured intervertebral disc and endplates as well as the restoration process of the PVO lesion, which may result in the persistent elevated ESR and VAS score of back pain. The values of ESR, CRP, and VAS score of back pain at the time of discontinuation of parenteral antibiotics or switching to oral antibiotics were 42.86 ± 24.05 mm/h, 0.91 ± 1.18 mg/dL, and 4.05 ± 1.07, respectively. There were no statistically significant differences between the two groups. We think that the values of ESR, CRP, and VAS score of back pain are meaningful and can help assess therapeutic response and decide regarding discontinuation of parenteral antibiotics or changing to oral antibiotics.

The recurrence rates reported in previous literatures have varied greatly. McHenry et al. [19] reported a recurrence rate of 14% in 253 patients, Park et al. [10] of 9.9% in 314 patients, and Kim et al. [18] of 6.6% in 151 patients. Unfortunately, data to guide the optimal duration of antibiotic therapy related to the recurrence of PVO are insufficient [7, 20–23]. Some studies reported that less than 6-8 weeks of antibiotic therapy may be associated with an increased risk of recurrence [18, 19, 24, 25]. Compared to the results of many previous literatures, our results showed relatively longer duration of antibiotics and lower recurrence rate of 6.8%. Kim et al. [18] reported a similar recurrence rate of 6.6% with an average of more than 100 days of the total antibiotics. Based on these results, we expect prolonged antibiotic therapy to be helpful in preventing recurrence. As mentioned above, culture-negative PVO shows fewer infectious signs and lower values of inflammatory markers, which may also be associated with a shorter duration of parenteral antibiotic therapy than in culture-positive PVO. However, as higher CRP level is usually associated with a higher risk of treatment failure in PVO, switching from parenteral antibiotics to oral antibiotics may be favorable in cases with lower CRP level [18, 26]. We think that the assessment of therapeutic response based on laboratory data allows for shortening the duration of parenteral antibiotics and hospital stay with favorable outcomes, particularly, under an environment with low rates of causative bacterial identification. However, we could not clearly explain the reasons for the frequent use and longer duration of oral antibiotics in culture-negative PVO. Considering the similar recurrence rate between the two groups, oral antibiotics are expected to prevent the recurrence despite the relatively short period of parenteral antibiotics in culture-negative PVO.

There were several limitations in this study. First, it was a retrospective study with relatively small number of participants, which may have resulted in an underpowered statistical analysis. Second, the sample size of patients who experienced recurrence was small, and thus the statistical analysis of several factors related to recurrence was impossible. Third, false-positives in the assessment of the therapeutic response requiring additional antibiotic therapy could not be accurately identified, which could have led to the unnecessary use of antibiotics. To overcome these limitations, further studies with a larger number of participants under a prospective design are required.

## Conclusion

Culture-negative PVO required a shorter duration of parenteral antibiotics and enabled changing to oral antibiotics compared to culture-positive PVO in terms of achieving similar outcomes. Discontinuation of parenteral antibiotics or switching to oral antibiotics can be considered based on the values of ESR, CRP, and VAS score for back pain. The presence of epidural abscess was the most significant factor for the identification of causative bacteria in PVO, which is associated with obtaining sufficient proper specimen via surgical culture.

## Data Availability

The datasets during and/or analysed during the current study are available from the corresponding author on reasonable request.

## Abbreviations

PVO: Pyogenic vertebral osteomyelitis
ESR: Erythrocyte sedimentation rate
CRP: C-reactive protein
VAS: Visual analogue scale
